# Evaluation of measures related to dosimetric uncertainty of VMAT plans

**DOI:** 10.1002/acm2.13862

**Published:** 2022-12-15

**Authors:** Julia Götstedt, Anna Karlsson, Anna Bäck

**Affiliations:** ^1^ Department of Radiation Physics Institute of Clinical Sciences Sahlgrenska Academy University of Gothenburg Gothenburg Sweden; ^2^ Department of Therapeutic Radiation Physics Medical Physics and Biomedical Engineering Sahlgrenska University Hospital Gothenburg Sweden

**Keywords:** dosimetric uncertainty, quality assurance, VMAT

## Abstract

Dosimetric uncertainty is most often not included in the process of creating and selecting plans for treatment. Treatment planning and the physician's choice of treatment plan is instead often based only on evaluation of clinical goals of the calculated absorbed dose distribution. Estimation of the dosimetric uncertainty could potentially have impact in the process of comparing and selecting volumetric modulated arc therapy (VMAT) plans. In this study, different measures for estimation of dosimetric uncertainty based on treatment plan parameters for plans with similar dose distributions were evaluated.

VMAT plans with similar dose distributions but with different treatment plan designs were created using three different optimization methods for each of ten patient cases (tonsil and prostate cancer). Two plans were optimized in Eclipse, one with and one without the use of aperture shape controller, and one plan was optimized in RayStation.

The studied measures related to dosimetric uncertainty of treatment plans were aperture‐based complexity metric analysis, investigation of modulation level of multi leaf collimator leaves, gantry speed and dose rate, quasi‐3D measurements and evaluation of simulations of realistic delivery variations.

The results showed that there can be variations in dosimetric uncertainty for treatment plans with similar dose distributions. Dosimetric uncertainty assessment could therefore have impact on the choice of plan to be used for treatment and lead to a decrease in the uncertainty level of the delivered absorbed dose distribution. This study showed that aperture shape complexity had a larger impact on dosimetric uncertainty compared to modulation level of MLC, gantry speed and dose rate.

## 1 INTRODUCTION

External photon radiotherapy is planned to deliver a distribution of absorbed dose with a prescribed dose to the tumour and at the same time as low dose as achievable to the surrounding healthy tissue. Volumetric modulated arc therapy (VMAT) is a treatment technique, for which the beam aperture shaped by a multi leaf collimator (MLC), the dose rate, and gantry speed are modulated simultaneously to create a dose distribution to meet user‐defined dose objectives and constraints.^1^

Similar dose distributions can be generated by treatment plans of different designs. Differences between treatment planners, optimization methods, dose calculation algorithms and different tools within the treatment planning system (TPS) might affect the dosimetric uncertainty related to the generated treatment plans.^2^, ^3^ Treatment plans with more complex beam apertures, that is, small and/or irregular MLC openings, will lead to dosimetric uncertainties expressed as larger dose deviations between planned and delivered dose distributions.^4^, ^5^, ^6^ Dose deviations could be caused by dose calculation accuracy limitations which occur to a larger extent for smaller and/or more irregularly shaped beam apertures, for example because of difficulties in accurate modelling of the source size, or because of regions with a lack of charged particle equilibrium.^7^, ^8^ Dose distributions planned to be delivered by smaller and/or more irregular beam apertures are also more sensitive to delivery variations, for example, differences between planned and actual position for MLC leaves.^9^, ^10^ Differences between planned and delivered dose have also been reported to be related to the level of modulation, that is, MLC leaf speed and acceleration, variation of gantry speed and dose rate.^11^ Even though studies^11^, ^12^, ^13^, ^14^, ^15^, ^16^ have shown notable dose differences between planned and delivered dose distributions due to the design of the treatment plans, the choice of treatment plan is most often solely based on a clinical assessment of the calculated dose distribution without considering the treatment plan design and the effect it might have on the dose uncertainty.^17^

There are different methods that can be integrated in the optimization process to lower the level of plan complexity with respect to beam aperture shape and size. A tool that is commonly available in commercial treatment planning systems is one that limits the number of monitor units (MU). Limiting the number of MU drives the beam aperture to be more open and can be used as an efficient tool to lower the plan complexity during optimization. However, the number of MU in itself does not correlate to dosimetric uncertainties and is therefore not an appropriate measure to estimate dosimetric uncertainty.^18^ Another method to lower the level of this type of plan complexity during optimization is the aperture shape controller (ASC) available in Eclipse™ TPS (Varian Medical Systems, version 15.5).^19^, ^20^ While MU and ASC are examples of tools in commercial TPSs that can be used to lower the treatment plan complexity, there are, to our knowledge, no commercially available tools for a final treatment plan complexity analysis to facilitate the optimal choice of alternative VMAT plans.

A common method for estimating dose discrepancies between planned and measured dose distributions, that is, dosimetric uncertainties, is to do pre‐treatment measurement‐based quality controls (QC). Measurements are suffering from inherent uncertainties and the QC result depends on the choice of measurement evaluation method.^12^ It has been shown that deliberately introduced dosimetric deviations are not always detected using common measurement‐based QC methods.^4^, ^12^, ^13^, ^14^ A measurement‐based QC method is time‐consuming, resource demanding and will provide information very late in the chain of treatment preparation. Furthermore, a pre‐treatment measurement is often conducted at a single occasion and the QC result will only reflect eventual delivery issues for that specific occasion of measurement.

One way to overcome uncertainties and limitations related to measurements could be to perform theoretical delivery simulations to evaluate the dosimetric impact of variations in mechanical delivery parameters. The drawback of such delivery simulations is that only uncertainties related to the delivery are included and uncertainties related to dose calculation accuracy limitations are not considered.

Evaluation of dosimetric uncertainties based on using complexity metrics have been suggested as a time efficient method. Aperture‐based complexity metrics, for example, the edge area metric (EAM),^4^ have been developed to consider uncertainties related to both delivery variations and dose calculation accuracy limitations, but do not include uncertainties related to dynamic parameters, that is, moving MLC leaves, rotating gantry and dose rate variations. Other metrics, so called modulation indices, for example, the modulation index (MI),^11^ have been suggested to score the complexity due to dynamic parameters, for example, MLC leaf speed and acceleration.

None of the above‐mentioned measures for estimation of dosimetric uncertainties take into account all possible causes of dosimetric uncertainties. Different measures give different type of information that could be more or less valuable when comparing and selecting VMAT plans to be used for treatment. A straight comparison of different measures related to dosimetric uncertainties for treatment plans of different designs has to our knowledge not yet been performed.

The aim of this study was to evaluate information gained by different measures related to dosimetric uncertainty and its value in the process of comparing and selecting VMAT plans to be used for treatment. The measures studied are aperture‐based complexity metric analysis, quasi‐3D measurements, delivery simulations, and assessment of treatment plan modulation.

## 2 METHODS

**FIGURE 1:**
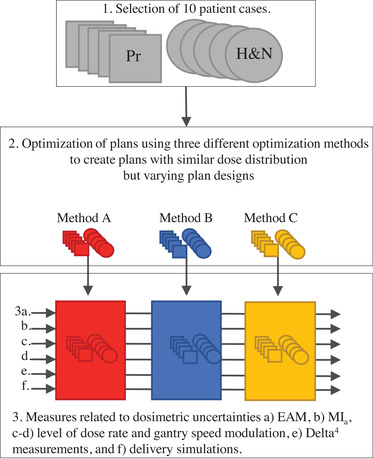
Flowchart illustrating the methodology. (1) Selection of plans planned for treatment of prostate (Pr) and head and neck (H&N) cancer, (2) optimization of plans using three different optimization methods (A, B, and C) to generate plans with similar dose distribution but varying plan designs, (3) analysing treatment plans by using different types of measures related to the dosimetric uncertainty (3a‐f).

The methodology of this study is illustrated in a flowchart in Figure 1.

### 2.1 Treatment plans

A set of VMAT plans was created using three different examples of optimization methods available at our department with the aim to create similar dose distributions but with a difference in plan design for each patient case. This study used anonymized CT data with associated structure sets of 10 patients (Figure 1, point 1). Five patients with tonsil cancer and five patients with prostate cancer (intermediate and high‐risk cancer) were consecutively, but excluding abnormal patient geometries, selected from our database of treated patients. The tonsil cancer patients had two separate target volumes with a prescribed absorbed dose of 68 Gy to the planning target volume (PTV) for the tumour region (PTV T) and 52.7 Gy to the lymph node regions (PTV N) to be delivered in 34 fractions. The prescribed absorbed dose to the prostate PTVs was 66 Gy in 22 fractions.

To be able to compare plans with similar dose distributions but of different treatment designs, we used three different optimization methods to create three different plans for each patient. The optimization methods are not of specific importance for this study and are referred to as method A, B and C. Two plans were optimized in Eclipse (Varian Medical Systems, version 15) without (method A) and with (method B) the use of ASC and one plan was optimized in RayStation (RaySearch Laboratories, version 8B) (method C) (Figure 1, point 2). The treatment planning goals used for head and neck (H&N) and prostate treatment planning within this study were based on local clinical practice, presented in Table 1.

**TABLE 1:** Treatment planning goals for head and neck and prostate treatments

Head and neck			Prostate		
Priority	Structure	Dose objective [%]	Priority	Structure	Dose objective [%]
1	Spinal cord	D_2%_ ≤ 68	1	CTVT	D_min_ ≥ 97
2	PTV T	D_98%_ ≥ 95	2	PTVT	D_98%_ ≥ 95
3	PTV N	D_98%_ ≥ 74	3	Rectum	V_90%_ ≤ 15
4	Parotid glands	D_mean_ ≤ 37 (both) or ≤ 29 (one)	4	Rectum	D_2%_ ≤ 100
5	Larynx	D_mean_ ≤ 59	5	Rectum	V_50%_ ≤ 50
5	Oral cavity	D_mean_ ≤ 35	6	Body	D_max_ ≤ 105
5	Upper esophagus	D_mean_ ≤ 59			
5	Mandible	D_2%_ ≤ 103			
5	Submandibular gland	D_mean_ ≤ 57			
5	Body	D_max_ ≤ 107			

*Note*: The clinically used treatment planning goals for head and neck include additional criteria for brain stem, pharynx and cochlea not used in this study. Similarly for prostate planning, criteria for the bladder and femoral heads are excluded in this study.

Abbreviation: CTV, clinical target volume, N, nodes, PTV, planning target volume, T, tumour.

Aperture shape controller was introduced as a tool in the photon optimizer algorithm in Eclipse to control and limit the complexity of the MLC beam apertures for VMAT treatment plans. It is implemented within the cost function of the optimization to penalize differences between adjacent MLC leaf tip positions. The magnitude of the penalty could be chosen by the user in five steps: very low, low, moderate, high and very high. The recommendation from the vendor is to use moderate or lower penalty for treatments which are expected to require a higher level of MLC modulation, for example, head and neck cases.^21^ The VMAT plans created in this study were optimized with the ASC set at moderate for the tonsil plans and very high for the prostate plans. The prostate plans were optimized using multi criteria optimization (MCO), available in both Eclipse and RayStation,^22^ except for plans created using ASC since it was not available for MCO. MCO generates a collection of pareto optimal treatment plans, that is, plans that cannot be improved for a specific objective without worsening it for another. The user can navigate in Pareto space to select the best treatment plan from the individual treatment purpose perspective.

The similarity of the dose distributions for each patient was evaluated visually, both regarding target dose coverage and sparing of normal tissue, as well as analytically, by comparing the dose volume histogram (DVH) metrics for the structures listed in Table 1. All plans were optimized to minimize hot and cold spots in the dose distribution. The dose calculation algorithms used were the analytical anisotropic algorithm (AAA version 15, 0.25 cm dose calculation grid) in Eclipse and collapsed cone (CC version 5.0, 0.25 cm dose calculation grid) in RayStation. All plans were designed with two full arcs, six MV and planned for a TrueBeam treatment machine equipped with the Millennium MLC (Varian Medical Systems). Each treatment arc was divided into 178 or 180 evenly distributed control points for treatment plans, created using Eclipse or RayStation, respectively. None of the plans created in this study have been used for treatment.

### 2.2 Evaluation of differences in dosimetric uncertainty

The dosimetric uncertainty of the optimized treatment plans were estimated by different measures: aperture‐based complexity metric analysis, assessment of treatment plan modulation, quasi‐3D measurements, and delivery simulations (Figure 1, point 3).

#### 2.2.1 Beam aperture and modulation evaluation

The aperture‐based complexity metric EAM was used to score aperture shape complexity of VMAT plans on a control point level.^4^, ^23^ EAM quantifies the extent of aperture edge in relation to the open part of the beam on a finite scale (0–1), where an EAM score of 1 means that the total aperture is within the complex region defined around the MLC edge. In this study, this complex region was defined to be 2.5 mm on both sides on the MLC edge since this setting has been shown to best separate EAM scores for beam apertures of different complexity.^23^

The modulation index (MI_a_) that was developed to evaluate MLC leaf speed and acceleration between subsequent control points^11^ was used in this study as a measure of the MLC modulation of the treatment plans. The differences in treatment plan design with respect to the modulation of dose rate and gantry speed were analysed. The dose rate and gantry speed were extracted for each control point and a two‐tailed Student's t‐test was used to compare differences in dose rate and gantry speed variations during treatment for plans created by the three optimization methods.

#### 2.2.2 Measurement‐based evaluation

All treatment plans were measured with the Delta^4^® cylindrical PMMA phantom (ScandiDos), inserted with two crossing detector planes consisting of a total of 1069 p‐type Si diodes. The dose accuracy of the device was specified by the manufacturer to 2% in a reference field relative to ionization chamber measurements.^24^ A correction for the daily machine output was applied based on a half arc field, rotation from 270 to 90 deg, with a 10 × 10 cm^2^ square aperture formed using the collimator jaws. All plans were consecutively measured once at the same occasion. All dose distributions (originally optimized in Eclipse and RayStation) were recalculated for the Delta^4^ phantom geometry in Eclipse and compared to the measured dose distributions. The Delta^4^ software provides a feature called optimized phantom position which suggests a virtual position shift of the phantom (three directions) to achieve the best dosimetric agreement. The mean value of the individually suggested shifts (based on 3%/1 mm gamma pass rate evaluation) for the 30 measurements was applied to align the calculated and measured dose distributions in the same way for all treatment plans prior to evaluation.

The difference between calculated and measured dose was evaluated for the diodes that reached more than a threshold dose of 20% of the calculated maximum dose. The fail rate [%] for the diodes within a global dose difference (DD) of 3%, normalized to the calculated maximum dose, was evaluated.

#### 2.2.3 Evaluation based on delivery simulations

During treatment delivery of VMAT plans, random variations occur because mechanical parts in the treatment machine are allowed to operate within tolerances specified by the manufacturer. Also, systematic offsets between planned and actual value for these machine parameters could be caused by uncertainties in the calibration procedures. Simulated treatment deliveries were performed as a method to study the dosimetric effect of offsets between planned and actual value for mechanical delivery parameters, that is, the position of the MLC leaves and collimator jaws as well as gantry and collimator angle. These delivery simulations made it possible to evaluate the impact on the dose distribution due to mechanical delivery offsets, without the limitations and uncertainties that a measurement would bring. For each simulation, these mechanical delivery parameters were altered in exported RP DICOM® files using an inhouse developed MATLAB® script. Altered DICOM files were re‐imported into Eclipse TPS (version 16) and new dose distributions using the altered parameter values were calculated.

In this study, the respective maximum offsets of the treatment machine parameters allowed in the simulations were carefully selected to generate realistic simulations. These maximum offsets were based on the technical specifications of the TrueBeam system,^25^ personal communication with Varian Medical Systems, and on data collected from the regular treatment machine quality controls at our department (Table 2). In each simulation, the applied offset was randomly generated according to the probability of a normal distribution centred at the planned parameter value and truncated at ±3σ, where 3σ corresponds to the maximum offset for that specific machine parameter (Table 2). The applied offsets for the positions of the upper and lower jaws, collimator angle and gantry angle were the same for all control points for a treatment plan. The simulated offset of the MLC leaves was derived in three steps. First, an offset for the MLC banks X1 and X2 was applied, the same shift for both banks and for all control points. Since the MLC calibration procedure is performed for the MLC banks separately, a further shift for one of the banks, the MLC bank X2, was applied. Additionally, the position for each MLC leaf was further shifted individually for all control points.

**TABLE 2:** Maximum offsets for the treatment machine parameters used for an upper and lower limit of the applied simulated delivery variations

Machine parameter	Per control point	Per plan
MLC bank X1 and X2		1 mm
MLC bank X2, offset from X1		0.3 mm
Individual MLC leaves	0.35 mm	
Upper jaws		2 mm
Lower jaws		1 mm
Collimator angle		0.6 degrees*
Gantry angle		0.3 degrees

*Note*: *The tolerance level for the collimator angle is the combined maximum offset based on the technical specification (0.5 deg) and an additional local tolerance (0.1 deg) that is used to admit delivery.

The simulations resulted in new calculated dose distributions for the altered DICOM files that were calculated based on the same CT data as the original VMAT plans. Nine simulations were performed for each of the 30 plans included in this study. The calculated dose distributions for these nine simulations were analysed together with the calculated dose distribution with no applied offsets. The dosimetric effect of the delivery variations was evaluated as the standard deviation (SD) of the ten calculated absorbed dose distributions on a voxel level in the patient geometry. Evaluation was done for voxels with a calculated dose higher than 20% of the prescribed dose. General differences in treatment plan robustness related to delivery uncertainties for the different optimization methods were evaluated in frequency histograms of the SD of the dose values. The effect of delivery variations on the dose distributions were also evaluated based on the SD of specified DVH metric values: D_2%_ for the spinal cord, D_50%_ for the PTVs, V_26Gy_ for the contralateral parotid gland and V_50%_ as well as D_2%_ for the rectum, for all included patients separately.

## 3 RESULTS

### 3.1 Treatment plans

**FIGURE 2:**
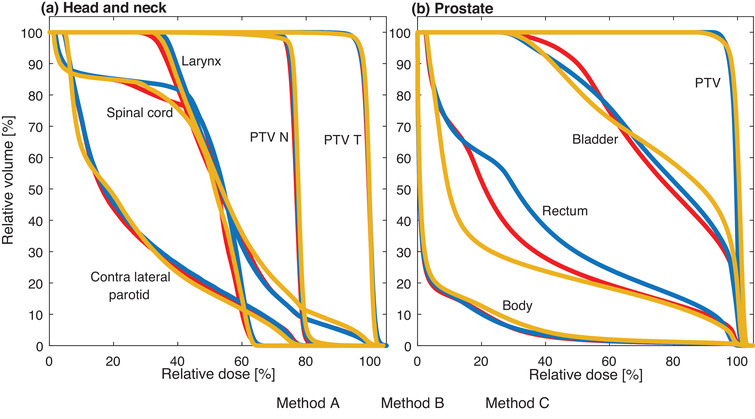
Cumulative dose volume histograms (DVH) for example cases of (a) a H&N treatment and (b) a prostate treatment for the three plans created with different optimization methods. The DVHs for the different optimization methods for the H&N cases included in this study was similar and the illustrated H&N case is representative for these plans. Some prostate cases resulted in a variation for the DVHs of the rectum structure and the illustrated prostate case was the case with the largest difference in rectum dose between the three optimization methods.

The highest prioritized dose volume objectives for the H&N treatment plans, that is, for spinal cord and PTV T, were achieved for all 15 plans. For the prostate treatment plans, all dose objectives were fulfilled for all 15 plans except for three plans which exceeded the D_2%_ dose objective for the rectum marginally with the highest value of 100.3%. The evaluated DVH metric values (Table 1) differed on average less than 1.5 (H&N) and 2 (prostate) local percentage points between the three versions of plans for each patient case. Typical variations in DVHs for one patient case of each treatment site are shown in Figure 2 as examples.

### 3.2 Evaluation of differences in dosimetric uncertainty

In general, the grading based on the results according to EAM and Delta^4^ measurement evaluations were higher for treatment plans optimized using method A (Table 3) which indicates a higher dosimetric uncertainty. However, the modulation level for these plans was lower compared to plans optimized using method C according to results of MI_a_ and dose rate and gantry speed variation evaluations. A more in‐depth presentation of the results can be found in following Sections 3.2.1–3.2.3.

**TABLE 3:** Schematic overview of the results of different measures related to dosimetric uncertainty for the groups of plans created using optimization methods A, B, and C (relative scale per column)

	Measures related to dosimetric uncertainty				
Gradation scale	EAM	MI_a_	Dose rate and gantry speed variations	Delta^4^ fail rate	Simulated delivery variations
Higher	A	C	C	A	A B
↑				B	
Lower	B C	A B	A B	C	C

#### 3.2.1 Beam aperture and modulation evaluation

**FIGURE 3:**
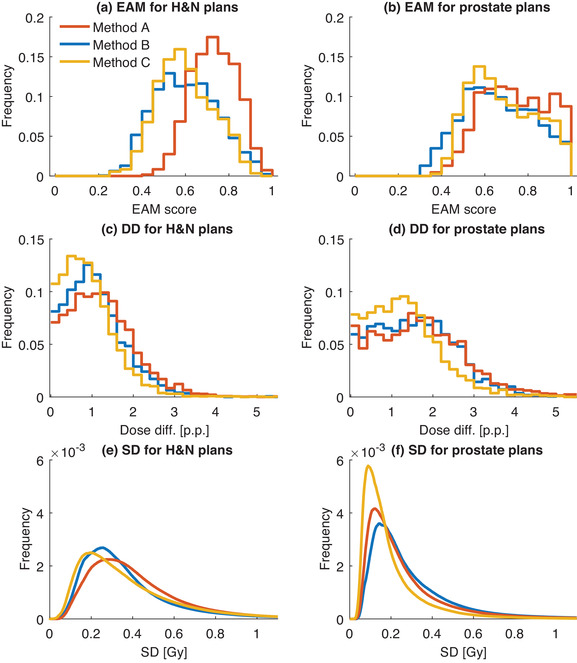
Frequency histograms for five double arc H&N and prostate VMAT plans optimized using three different methods A (red), B (blue), and C (yellow). (a‐b) Histograms for EAM score consisted of 1780 control points for plans created with methods A and B and 1800 control points for plans created with method C. (c‐d) Frequency histograms for the dose difference (DD) between calculation and Delta^4^ measurement for detector points. (e‐f) Histograms of standard deviation (SD) of dose in voxels for ten calculated dose distributions per plan with different simulated delivery variations.

A larger fraction of control points with higher EAM scores, indicating more complex apertures, was found for the treatment plans created using method A compared to the plans created using methods B and C (Figure 3a,b). This result was most expressed for the H&N cases (Figure 3a). A larger fraction of control points with higher EAM scores was found for prostate plans compared to H&N plans for all optimization methods.

**FIGURE 4:**
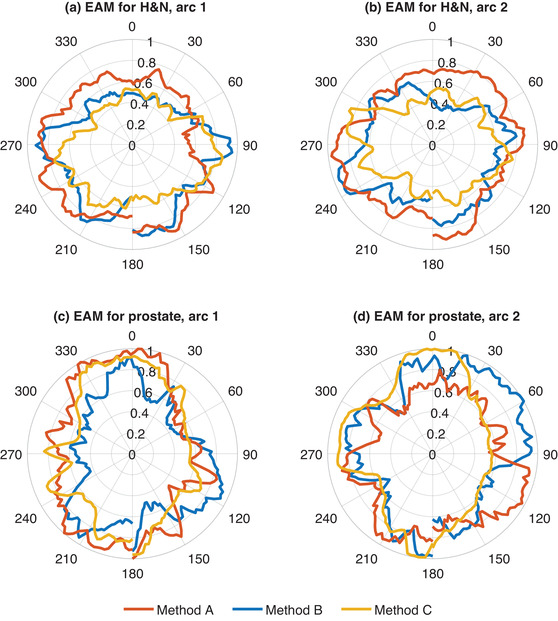
Polar plots for EAM scores per gantry angle for one representative patient case of each diagnose, H&N and prostate (the same patient cases as presented in Figure 2). Plans were optimized using three different methods A, B, and C.

The polar plots of EAM per gantry angle for the H&N cases demonstrated that the EAM scores were higher in the majority of gantry angles for the plans created with method A compared to the other optimization methods (Figure 4). This general pattern was not found in the polar plots for the prostate cases.

**FIGURE 5:**
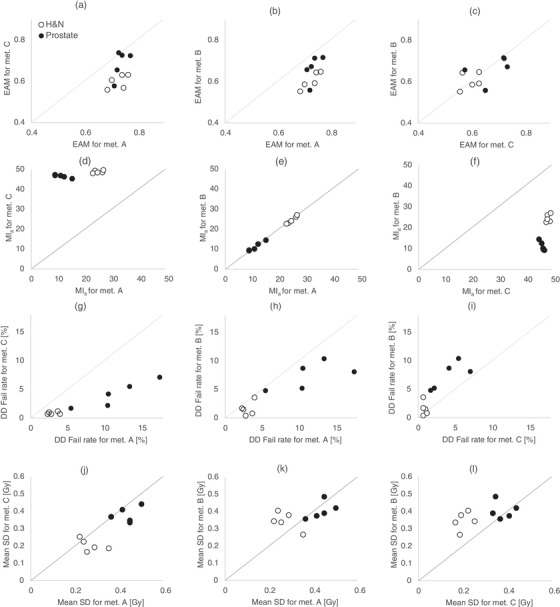
One to one comparisons of the different optimization methods (A, B and C) for each patient case. Open circles for H&N plans and filled circles for prostate plans. The mean of EAM scores per control point for a total plan compared for (a) optimization method C versus A (b) method B versus A and (c) method B versus C. The corresponding result of the MI_a_ score comparisons is shown in (d), (e), (f). Comparison of the 3% dose difference (DD) fail rate for Delta^4^ measurements is shown in (g), (h), (i). Delivery simulation results expressed as the mean of the standard deviation (SD) of dose values in voxels (Gy) for ten simulations of dose distributions for each treatment planare shown in (j), (k), (l).

The mean values of the EAM scores for all control points per plan were, except for one case, higher for the plans created with method A compared to the plans created with method B and C (Figure 5a,b). There was no systematic difference between total plan mean EAM for plans created with methods B and C (Figure 5c).

The MI_a_ score was systematically higher for the plans created with method C compared to the plans created with methods A and B (Figure 5d–f), indicating higher MLC leaf speed and acceleration for method C plans. MLC modulation was scored similar for plans created with methods A and B according to the MI_a_ scores. A difference between the diagnose groups could be seen for the plans created with methods A and B, as the H&N plans had a higher MLC modulation according to the MI_a_ evaluation. This difference was not found for the plans created with method C.

**FIGURE 6:**
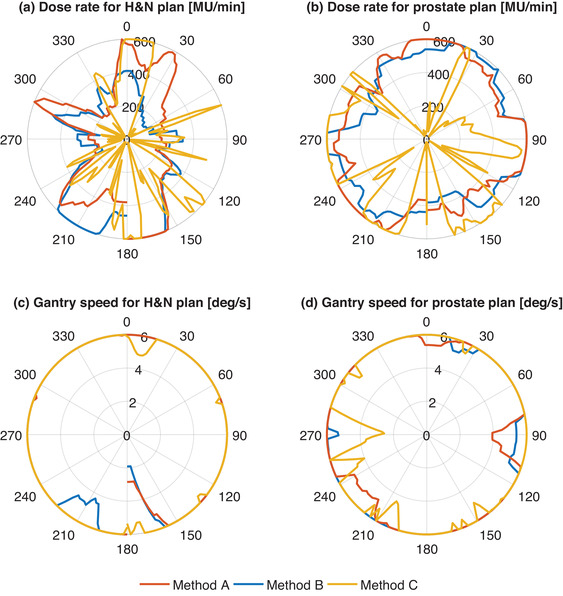
Dose rate (a, b) and gantry speed (c, d) variation as a function of gantry angle for a representative treatment arc for a H&N and a prostate case for the plans created using the three optimization methods A, B and C. The same arcs as shown in Figure 4a and c.

There was no statistical difference between the plans created with method A and B regarding the variation between control points for dose rate and for gantry speed (*p*‐value > 0.05). There was a statistical difference between the plans created with methods A and B compared to plans created with method C for both dose rate and gantry speed modulation for H&N and prostate plans (*p*‐value < 0.05), where the modulation was larger for the plans created with method C (Figure 6).

#### 3.2.2 Measurement‐based evaluation

The frequency histogram for the dose difference between calculation and measurement showed a larger fraction of evaluated measurement points with a smaller dose difference for plans created with method C compared to plans created with method A and B for both H&N and prostate treatment plans (Figure 3c,d).

The dose difference fail rate from the evaluation of the Delta^4^® measurements was systematically higher for the prostate compared to H&N treatment plans (Figure 5g–i). The DD fail rate for the treatment plans created with method A, for both H&N and prostate plans, were higher compared to plans created with the other optimization methods, similar to the EAM evaluation results. However, the measurement evaluation results indicate a higher fail rate also for plans created using method B compared to method C (Figure 5i), which was not seen in the EAM evaluations (Figure 3a,b, Figure 5c).

#### 3.2.3 Evaluation based on delivery simulations

A larger fraction of evaluated voxels with lower SD of dose values, indicating a higher delivery robustness, was found for the plans created with method C as the histograms were shifted towards lower values compared to plans created with methods A and B. This was most pronounced for the prostate cases (Figure 3e,f). This result was supported by the one‐to‐one comparisons (Figure 5j, l). No systematic differences regarding simulated delivery variations were found between the plans created with methods A and B (Figure 5k).

**FIGURE 7:**
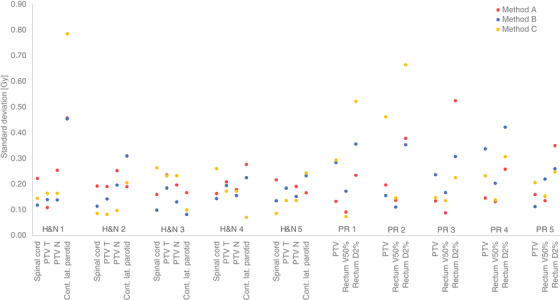
Standard deviation of some DVH metric values for ten dose distributions simulating delivery variations for each included head and neck (H&N) and prostate (PR) treatment plans optimized using three different methods A, B, and C.

The effect of delivery variations in specific DVH metric values was most often within 0.5 Gy (Figure 7). For the H&N plans, the majority of the evaluated DVH metric values resulted in larger SDs for the plans created with method A, that is, 13 of 20 evaluated metrics. However, the variations in DVH metric values were found to be irregular and patient case dependent.

## 4 DISCUSSION

Evaluation of VMAT plans with similar absorbed dose distributions showed a difference in measures related to dosimetric uncertainty between treatment plans of varying treatment plan design. The aperture shape complexity according to EAM scores was similar for plans created with method B and C and lower compared to the EAM scores for plans created with method A (Table 3). The measurement‐based evaluation was in agreement to the EAM scores but furthermore also separated the plans optimized with method B from the plans optimized with method C. The delivery simulations did not separate methods A and B but indicated, in agreement with the measurement‐based evaluation, a lower dosimetric uncertainty for method C. The plans optimized with method C had higher MLC modulations, according to MI_a_ scores, and larger variations of the dose rate and gantry speed. It has been reported by others that higher modulation according to MI_a_ is correlated to higher dosimetric uncertainties.^11^ This was not found in our study, as supported by both measurement‐based evaluations and delivery simulations. The fact that Delta^4^ measurements showed a lower fail rate for plans created with method C compared to plans created with method A, and for the majority of plans created with method B, indicates that dosimetric uncertainty is more affected by the complexity of the beam aperture shape than by modulation of the MLC, gantry speed and dose rate.

Although it was not an aim of this study to compare specific optimization methods or TPSs, the results of this study showed that treatment plans created in RayStation (method C) had a generally lower level of dosimetric uncertainty compared to plans created in Eclipse (method A and B) according to the results of Delta^4^ measurements and delivery simulations. A general difference between the optimization strategies in the two different TPSs was found to be that RayStation generated plans with higher MLC leaf speed and acceleration (i.e., a higher MI_a_) and larger variations in dose rate and gantry speed. However, any alternative strategies for treatment planning or the possibility to change any settings in the TPS was not studied and therefore a more general conclusion on differences between the optimization methods cannot be made based on this work. The treatment plan design was in this study dependent on the treatment planning system, but differences between treatment machines and strategies used by the treatment planner do also have impact on plan design.^26^

Treatment plans created in Eclipse with ASC (method B) were found to be less complex regarding aperture shape compared to the plans created in Eclipse without ASC (method A) according to generally lower EAM scores on control point and treatment plan level. A decrease in aperture shape complexity for plans created with ASC compared to without has also been shown in other studies.^20^, ^27^ Further, Delta^4^ dose difference fail rate was also lower for treatment plans created in Eclipse using ASC compared to not using ASC. Our results show that optimization including ASC within Eclipse could lower the treatment plan complexity and dosimetric uncertainty without compromising the dose distribution for the plans included in this study. What the clinical gain will ultimately be for the specific patient when choosing a treatment plan with lower aperture shape complexity was not studied within the scope of this work but will be a subject for further studies within our research group.

The results for the two groups of different diagnoses, prostate, and H&N, were separated by the measurement‐based evaluation method and when evaluating the uncertainties due to delivery variations using delivery simulations with a higher fail rate and SD for the prostate cases. The results shown in the EAM histograms (Figure 3) indicate that prostate plans had a larger amount of control points in the higher range of EAM scores, but a separation between the two diagnose groups was not as obvious for the evaluation of the mean EAM evaluation (Figure 5). The higher aperture shape complexity and dosimetric uncertainty for prostate plans could partly be explained by smaller beam apertures compared to the H&N plans, which has been reported and discussed in one of our earlier publication.^23^ The smaller beam apertures will introduce more uncertainties as a relatively larger part of the beam will be close to the aperture edge where most of the uncertainties manifest. Furthermore, the prostate plans included in this study were planned with two full arcs which allows the use of smaller and/or more irregular MLC apertures compared to a plan with only one arc which is sometimes used in other studies.^28^ The MI_a_ evaluation separated the two groups of different diagnoses for the plans created in Eclipse, scoring a higher modulation for the H&N plans. This might be due to larger MLC travel distances because of the larger target volumes for H&N compared to prostate. This was, however, not seen for the plans created in RayStation that had generally higher MI_a_ for all cases compared to the plans created in Eclipse.

The different measures related to the dosimetric uncertainty used in this study concern different aspects of uncertainties and can therefore not be expected to give the same results. However, the combination of the different analyses can give a more complete picture of the uncertainty level of the treatment plans. Delta^4^ measurements and EAM analyses are methods which, to different degrees, include an estimate of the dosimetric uncertainty related to both absorbed dose calculation and treatment delivery. Delta^4^ measurement gives information about the position and extent of dose differences between planned and delivered dose but is limited to the geometry of the phantom. Also, the uncertainty due to the modulation of gantry speed, dose rate and MLC leaves, will be included in a measurement‐based analysis. These modulation parameters are not considered in the EAM analysis but the robustness to delivery variations is embedded in the theory behind EAM. However, the delivery variation and the parameter modulation are sub‐optimally considered in the measurement‐based evaluation since the delivery uncertainty will only be based on one delivery occasion.

The method to estimate the dosimetric uncertainty based on simulations of delivery variations have the advantage to enable evaluations of where dosimetric differences occur within the three‐dimensional patient geometry and could be directly linked to clinical relevance. However, the method of delivery simulations only includes analyses of the robustness to delivery variations and does not consider uncertainties related to absorbed dose calculation limitations in the TPS or the dynamic nature of the plan. The position of dose distribution uncertainties originating from both calculation and delivery uncertainties in the patient geometry cannot be evaluated by any of the methods included in this work. A first step towards developing such an evaluation method has been suggested in a parallel work by our group.^29^ A few cases of delivery simulation evaluations resulted in the highest deviations for some of the evaluated DVH metric values in structures for RayStation plans compared to Eclipse plans, despite that most of the results of this work indicate that plans created in RayStation have a lower dosimetric uncertainty level compared to plans created in Eclipse. For example, larger deviations were found for the dose to the spinal cord for the patient cases H&N3 and H&N4 and the dose to target and rectum for the patient cases P1 and P2 (Figure 7). This emphasizes that it is the combination of the treatment plan design and the patient geometry that is of importance when assessing the clinical relevance of the uncertainty of the treatment.

## 5 CONCLUSION

Treatment plans with similar dose distributions can have different inherent dosimetric uncertainties. The dosimetric uncertainty can be lowered when using an alternative treatment planning strategy. Including assessment of dosimetric uncertainty in the process of comparing and selecting VMAT plans to be used for treatment can lead to a more optimal choice of a plan with lower uncertainties compared to making decisions based only on the clinical trade‐offs for a calculated absorbed dose distribution. This study showed that aperture shape complexity had a larger impact on dosimetric uncertainty compared to modulation level of MLC, gantry speed and dose rate.

## AUTHOR CONTRIBUTION

All authors have contributed equally through joint discussions to the study design, the analysis and interpretation of the study results. The first author has performed the most work producing the data with supervision of the co‐authors. The text of the submitted manuscript has been finalized by all authors together. All authors approve and are well versed in the content of the final version of the manuscript.

## CONFLICT OF INTEREST

There are ongoing collaborations between our department and Varian Medical Systems which this project is not part of. Therefor, the authors have no conflict of interest to disclose.

## ETHICS STATEMENT

Research collaboration is ongoing between our department at the Sahlgrenska University Hospital and Varian Medical Systems in projects other than this.
